# Supplementary material for: Single-cell genomics improves the discovery of risk variants and genes of atrial fibrillation
Source: Nat Commun. 2023 Aug 17;14:4999. doi: 10.1038/s41467-023-40505-5 (PMC10435551; doi:10.1038/s41467-023-40505-5)
Supplement: Supplementary file 1 — Supplementary Information [file 41467_2023_40505_MOESM1_ESM.pdf]

Supplementary Information

Table of Contents

Supplementary Figures 2
Supplementary Notes 17
Supplementary References 19

Supplementary Figures

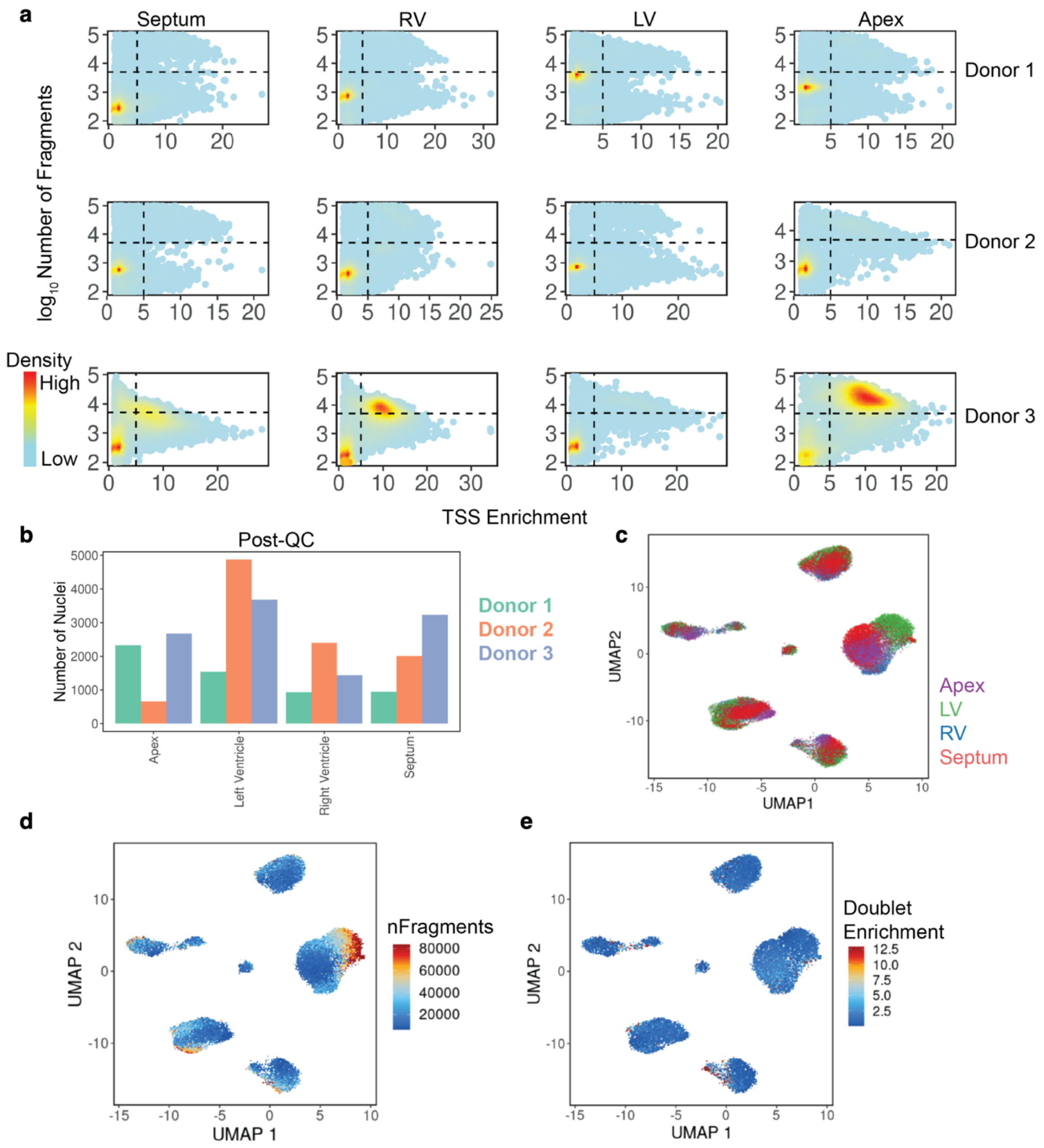

Supplementary Fig. 1: QC metrics for scATAC-seq samples across samples, donors, and clusters. a, Number of reads vs. TSS enrichment for all included libraries. Cells with more than 5,000 unique fragments and a TSS enrichment score above 5 were included. **b**, Number of cells passing QC per region and donor. **c**, Anatomic location, **d**, Number of fragments, **e**, Doublet enrichment scores overlaid on two-dimensional representation of cells using UMAP.

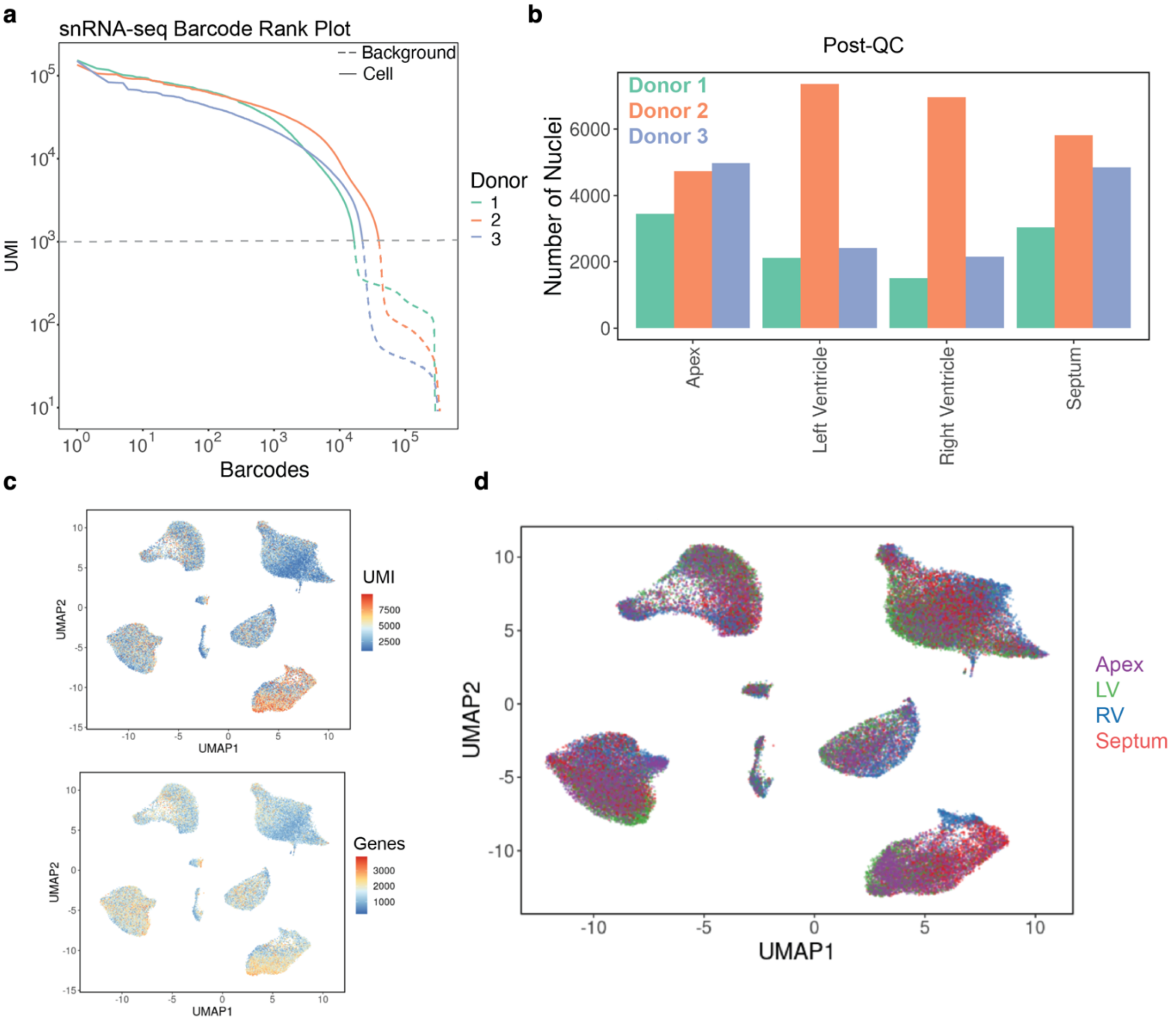

Supplementary Fig. 2: QC metrics for snRNA-seq samples across samples, donors, and clusters. a, UMIs vs barcodes ranked by UMI count. Shown are combined samples for each donor. Cells with more than 1,000 UMIs were included. **b**, Number of nuclei passing QC per region and donor. **c**, Number of UMIs (top) and detected genes and **d**, Anatomic location overlaid on two-dimensional representation of nuclei using UMAP.

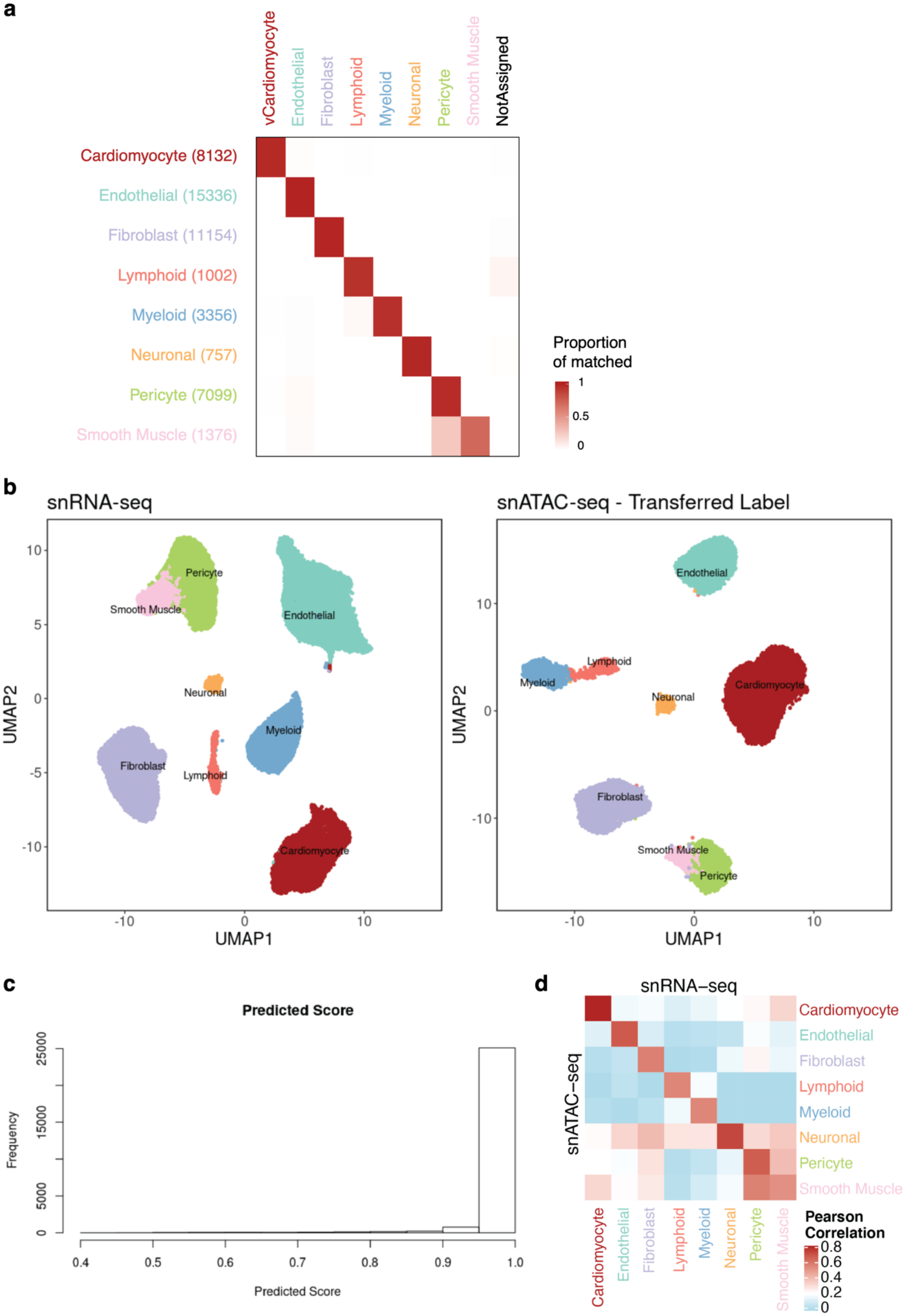
a
vCardiomyocyte
Endothelial
Fibroblast
Lymphoid
Myeloid
Neuronal
Pericyte
Smooth Muscle
NotAssigned
Cardiomyocyte (8132)
Endothelial (15336)
Fibroblast (11154)
Lymphoid (1002)
Myeloid (3356)
Neuronal (757)
Pericyte (7099)
Smooth Muscle (1376)
Proportion of matched
1
0.5
0
b
snRNA-seq
snATAC-seq - Transferred Label
Pericyte
Smooth Muscle
Endothelial
Neuronal
Myeloid
Fibroblast
Lymphoid
Cardiomyocyte
UMAP1
UMAP2
c
Predicted Score
Frequency
0
5000
15000
25000
0.4
0.5
0.6
0.7
0.8
0.9
1.0
d
snRNA−seq
snATAC−seq
Cardiomyocyte
Endothelial
Fibroblast
Lymphoid
Myeloid
Neuronal
Pericyte
Smooth Muscle
Pearson Correlation
0.8
0.6
0.4
0.2
0

Supplementary Fig. 3: Label transfer of cell-type labels from snRNA-seq to scATAC-seq using Seurat anchor transfer. a, Comparison of cell-type annotation in our study to labels transferred from Heart Cell Atlas, Litvinukova *et al.*[1]. Rows are cluster labels in our study with the number of cells indicated for each cell type in brackets. Columns are labels from Litvinukova *et al*. Shown are the proportions of cells per cluster (row) that are annotated with a given label in Litvinukova et al. Of the 49539 cells in our study, 48359 (97.6%) were annotated the same labels with Litviňuková's. 1180 (2.4%) had discordant cell type levels and 349 of them were not matched and fell into the "NotAssigned" category. **b**, Heart cells profiled by snRNA-seq (left) and scATAC-seq (right) embedded in two dimensions (UMAP). Cluster labels determined based on transcriptional signatures of snRNA-seq data (left) were transferred to scATAC-seq dataset (right). **c**, Distribution of cluster label prediction scores for each cell in the scATAC-seq dataset. **d**, Pairwise correlations (Pearson's r) between gene scores (scATAC-seq) and transcript levels (snRNA-seq) for differentially expressed genes between cell types using aggregated data from all cells within a cluster (pseudo-bulk).

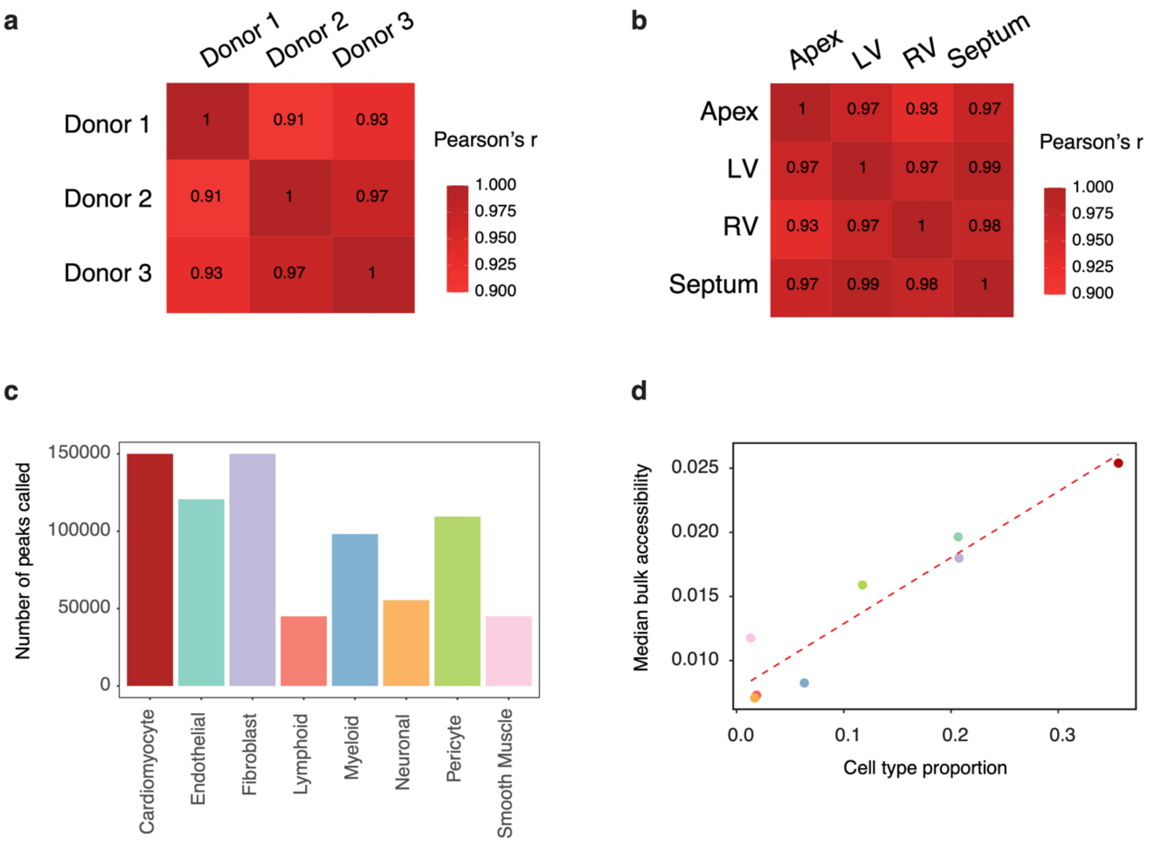

Supplementary Fig. 4: Comparison of OCRs between individuals and across cell types. a, Pairwise correlations (Pearson's r) of chromatin accessibility between individual donors. The comparisons were made between pseudo-bulk samples aggregating all cells from each donor. **b**, Pairwise correlations (Pearson's r) of chromatin accessibility between sampling locations. The comparisons were made between pseudo-bulk samples aggregating all cells from each location (including all three donors). **c**, Number of detected peaks in each cluster/cell type using MACS2. **d,** Correlation between the proportion of a cell type in the heart (X-axis) and the median accessibility, at the bulk level, of all peaks found in that cell type (Y-axis). Each dot represents one cell type, using the color scheme of Panel c). To define the median accessibility signal of a cell type, we first obtained the accessibility signal of any peak from the pseudo-bulk data, pooling all cells. We then took the median of the accessibility of all peaks called in any given cell type.

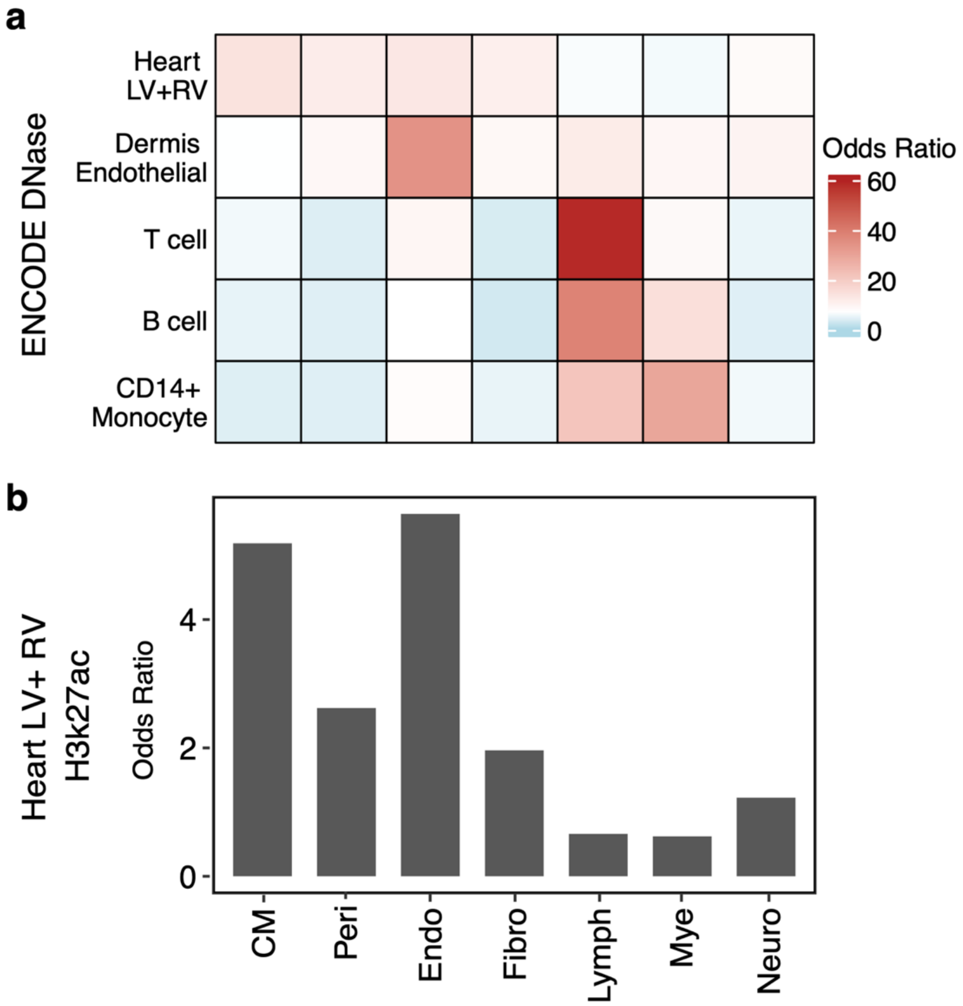

Supplementary Fig. 5: Comparison of open chromatin regions between the current study and ENCODE DNase hypersensitivity sites (DHS) from the indicated tissues (a) and ENCODE H3K27ac peaks in the heart (b). Abbreviations for cell-types: CM = Cardiomyocyte, Peri = Pericyte, Endo = Endothelial, Fibro = Fibroblast, Lymph = Lymphoid, Mye = Myeloid, Neuro = Neuronal.

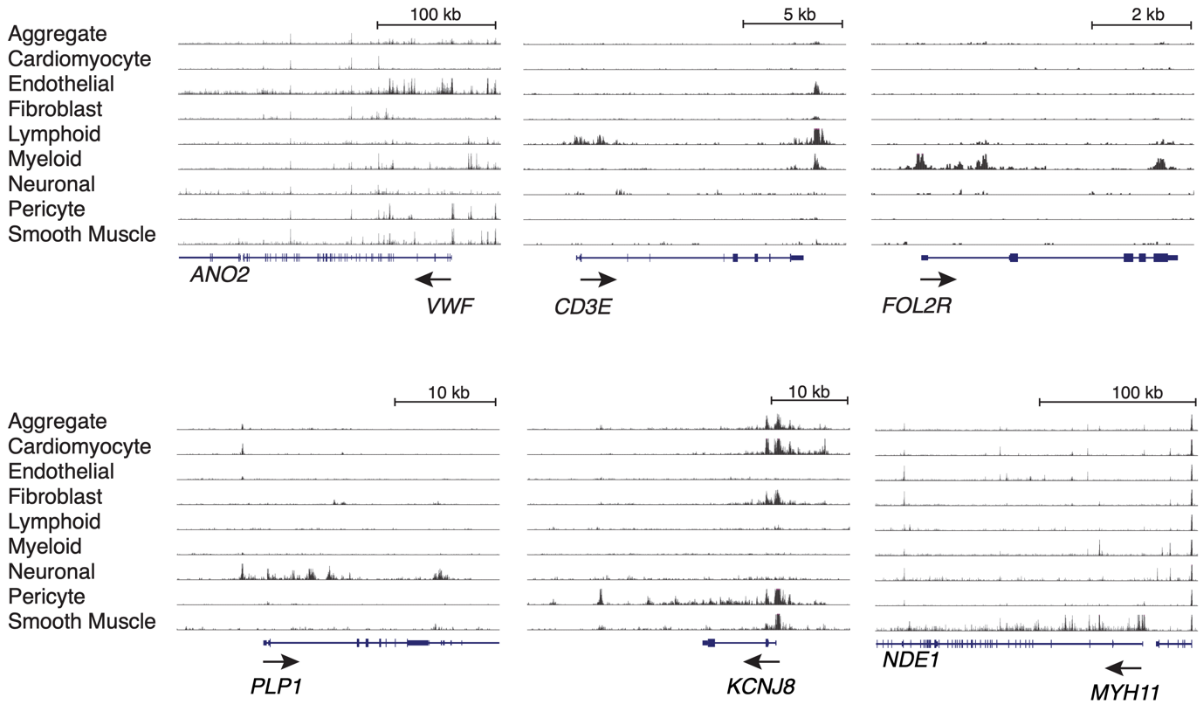

Supplementary Fig. 6: Genome browser track plots of chromatin accessibility at marker genes across cell types, as in Figure 2b.

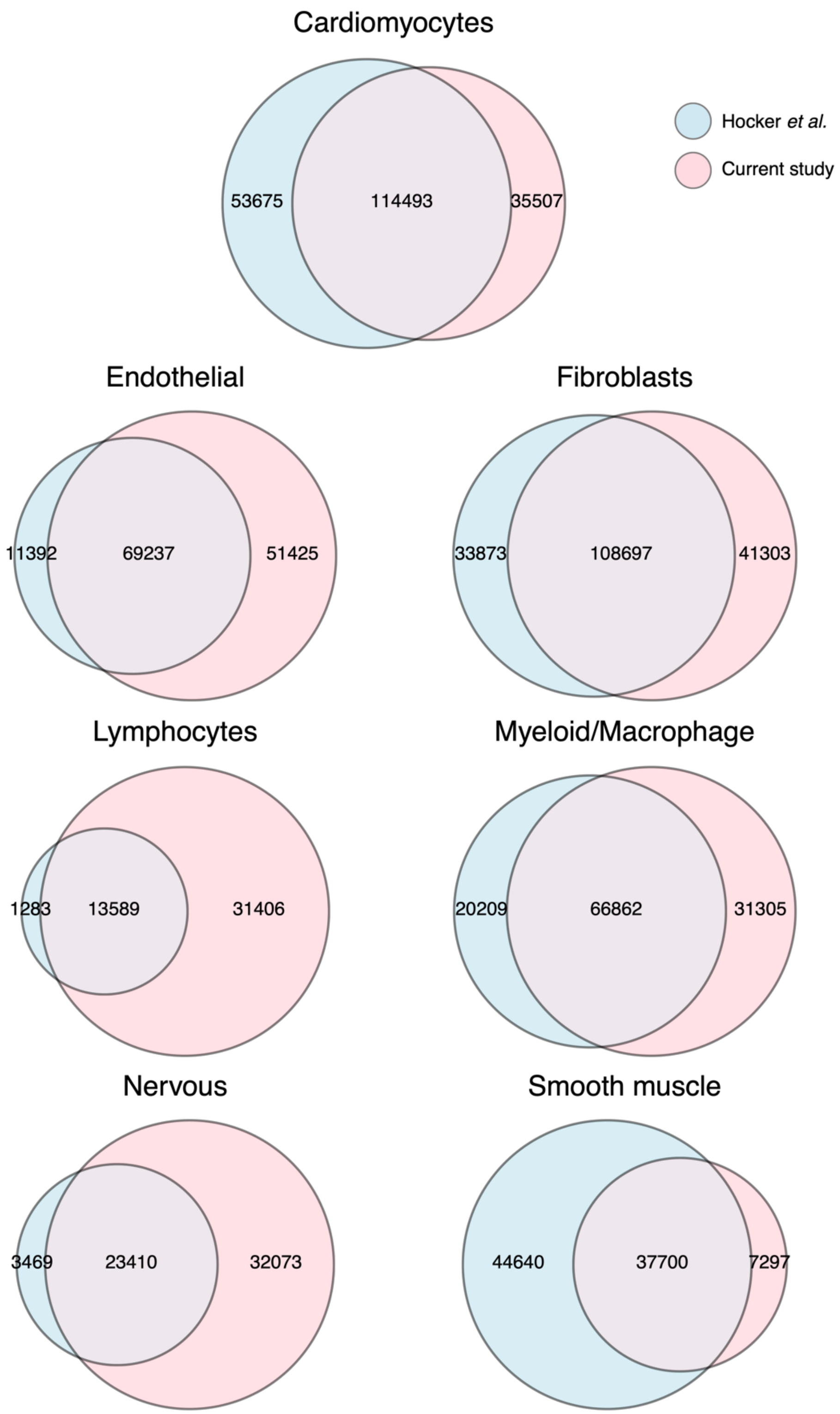

Supplementary Fig. 7: Comparison of Peaks identified by Hocker *et al.* and this study. The numbers of peaks in the overlapped areas of the Venn Diagrams are from this study. The numbers of overlapped peaks from Hocker *et al.* are similar.

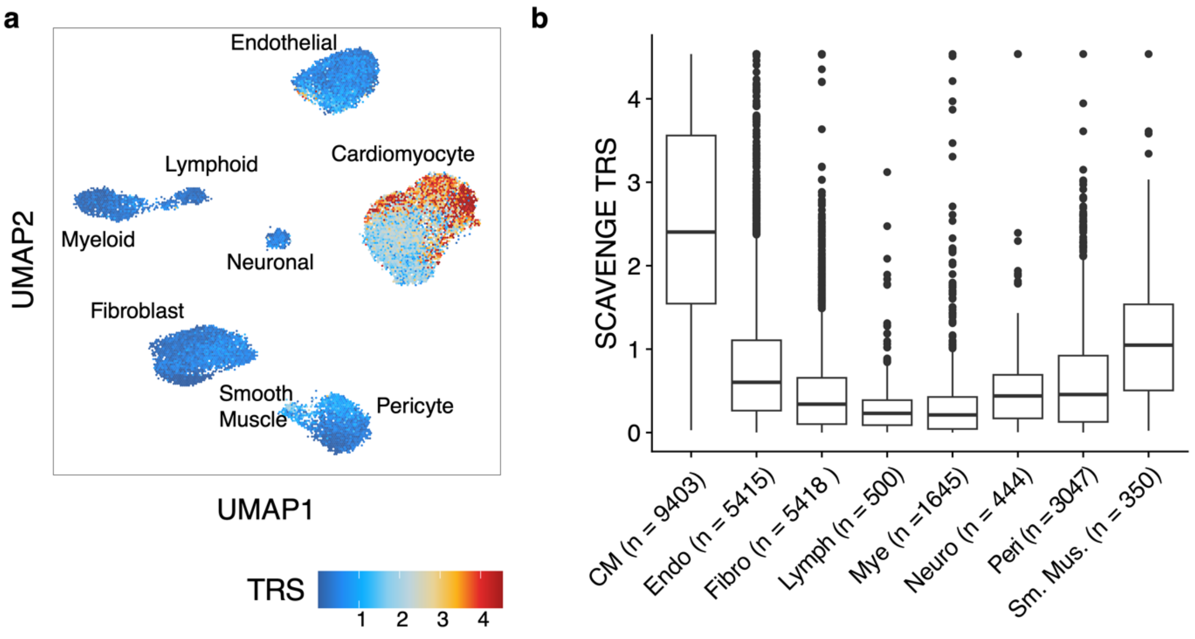

Supplementary Fig. 8: TRS scores from SCAVENGE are highest in Cardiomyocytes. **a**, TRS for each cell plotted on UMAP embedding. **b**, Distribution of TRS for each of our cell type clusters. The center line of a box represents the median; the lower and upper hinges of a box correspond to the first and third quartiles; the upper/lower whisker extends from the hinge to the largest/smallest value no further than 1.5 * inter-quartile range from the hinge. Abbreviations for cell-types: CM = Cardiomyocyte, Endo = Endothelial, Fibro = Fibroblast, Lymph = Lymphoid, Mye = Myeloid, Neuro = Neuronal, Peri = Pericyte, Sm.Mus. = Smooth Muscle.

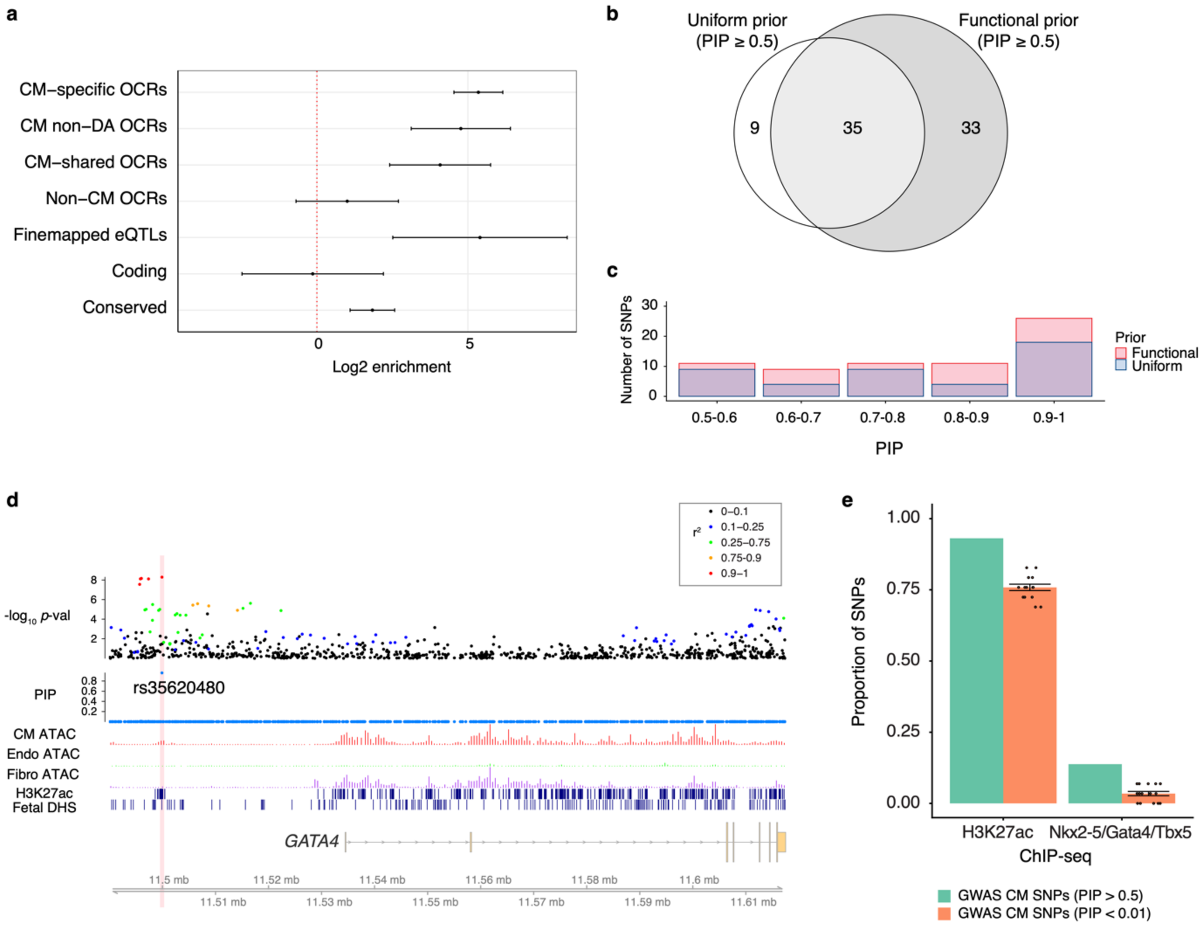

Supplementary Fig. 9: Enrichment analysis and function of putative AF risk variants. a, Log2 fold enrichment of AF risk variants in five functional categories, based on TORUS. Error bars represent 95% confidence intervals. The enrichment values were obtained in a joint model where all five annotations are included. The resulting variant probabilities from TORUS were used as priors for fine-mapping with SuSiE. **b,** SNPs with PIP >= 0.5 identified using functional prior vs. uniform prior **c,** Comparing PIP distributions between functional prior and uniform prior (only showing SNPs with PIP >= 0.5). **d**, Example of fine mapping with functional prior around *GATA4* locus. Abbreviations for cell-types: CM = Cardiomyocyte, Endo = Endothelial, Fibro = Fibroblast. **e**, Proportions of high and low confidence SNPs (based on fine-mapping) in H3K27ac ChIP-seq region in the human heart (ENCODE) and in Nkx2-5/Gata4/Tbx5 ChIP-seq regions identified in the mouse heart and lifted over to the human genome. n = 29 GWAS SNPs with PIP > 0.5 are in CM-specific OCRs, among those, 27 SNPs are in H3K27ac ChIP-seq regions, and 4 SNPs are in Nkx2-5/Gata4/Tbx5 ChIP-seq regions. For random controls, 15 sets of control SNPs were sampled from SNPs with PIP < 0.01 in CM-specific OCRs (n = 29 in each set to match the numbers of high confidence SNPs). Error bars represent mean +/- SE of the 15 control sets.

a

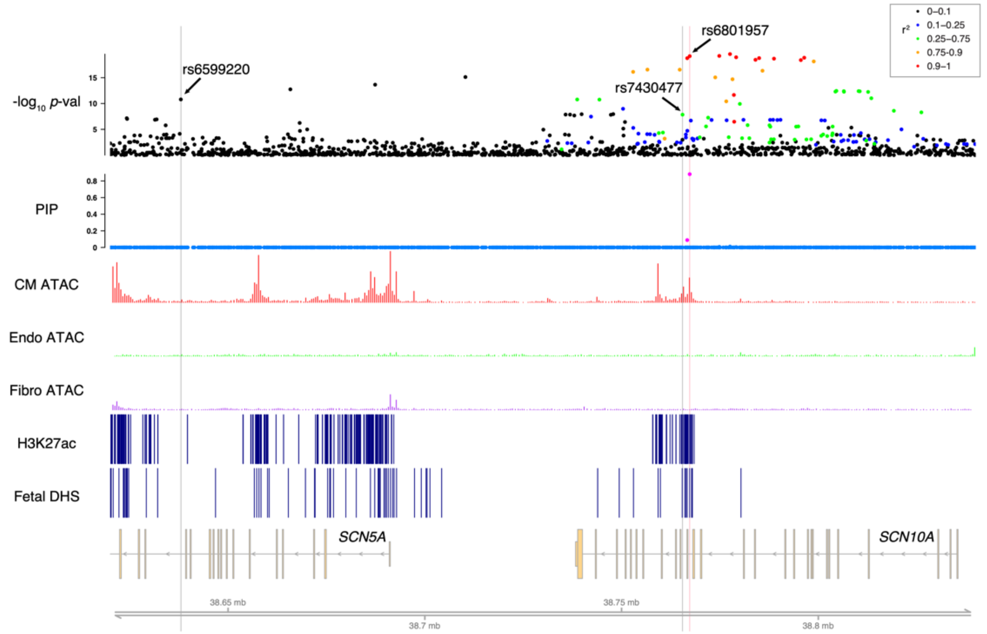

b

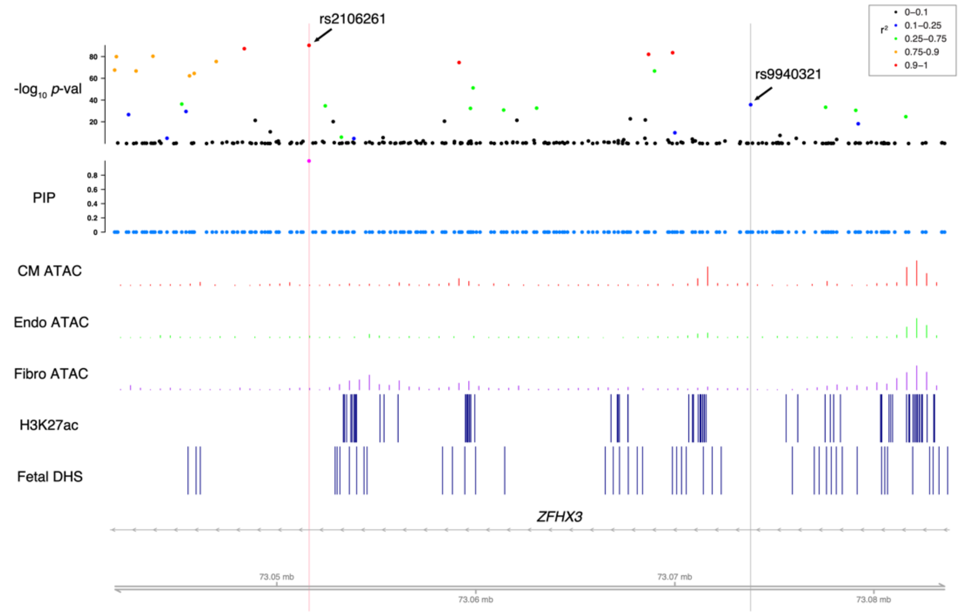

Supplementary Fig. 10: Examples of fine-mapping results for allele-specific variants from STARR-seq. For each locus, data tracks shown (from top to bottom) are: association with AF phenotype from GWAS; PIP from fine mapping taking into account OCRs and other functional categories; pseudo-bulk chromatin accessibility profiles for CMs (red), endothelial cells (green), and fibroblasts (purple); H3K27ac ChIP-seq and fetal DHS peak calls. Allele-specific variants from STARR-seq (van Ouwerkerk *et al., Circ. Res.* 2020) were highlighted in gray, and fine-mapped variants from the current study were highlighted in pink. Abbreviations for cell-types: CM = Cardiomyocyte, Endo = Endothelial, Fibro = Fibroblast.

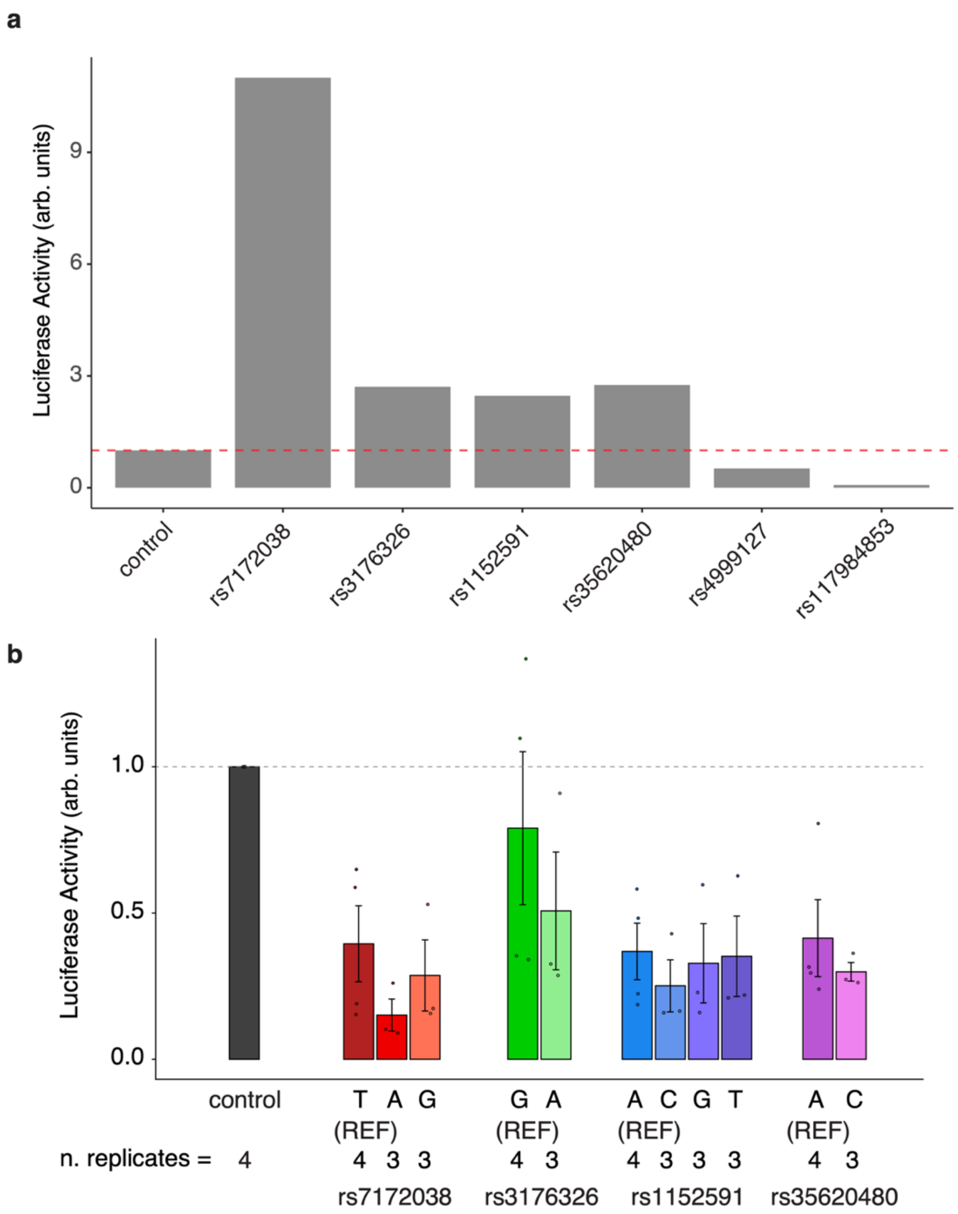

Supplementary Fig. 11: Assessing reporter gene activation for selected regions and variants. **a,** Result of initial screening of 6 candidate regions for reporter gene activity. Experiment was used to filter out regions or constructs that showed no activity before moving to measurements of allelic effects. Only a single replicate was obtained at this stage. **b,** Reporter activities in 3T3 fibroblasts of regions containing selected SNPs, with reference and alternative alleles (same regions as in Fig. 4j). Error bars represent mean +/- SE.

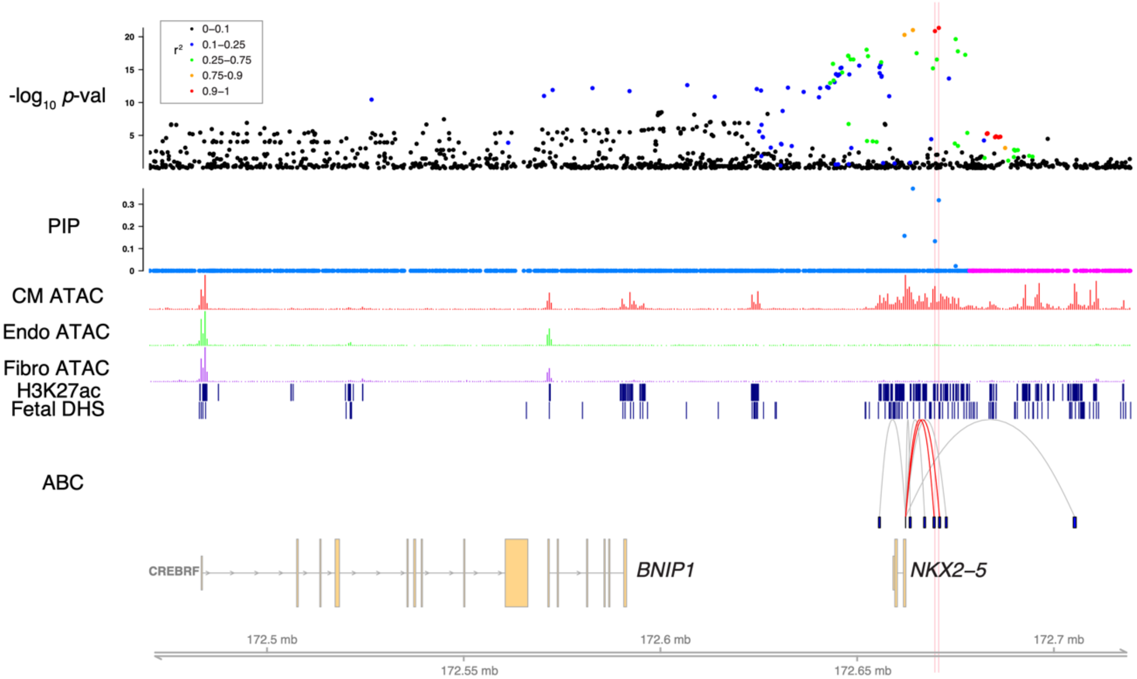

Supplementary Fig. 12: An example locus containing *NKX2-5* as the candidate gene (PIP = 0.99). Data tracks shown (from top to bottom) are: association with AF phenotype from GWAS; PIP from fine mapping taking into account OCRs and other functional categories; pseudo-bulk chromatin accessibility profiles for CMs (red), endothelial cells (green), and fibroblasts (purple); H3K27ac ChIP-seq and fetal DHS peak calls; interaction data from ABC scores from heart ventricle. Interactions anchored on the highlighted SNPs are highlighted in red. Abbreviations for cell-types: CM = Cardiomyocyte, Endo = Endothelial, Fibro = Fibroblast.

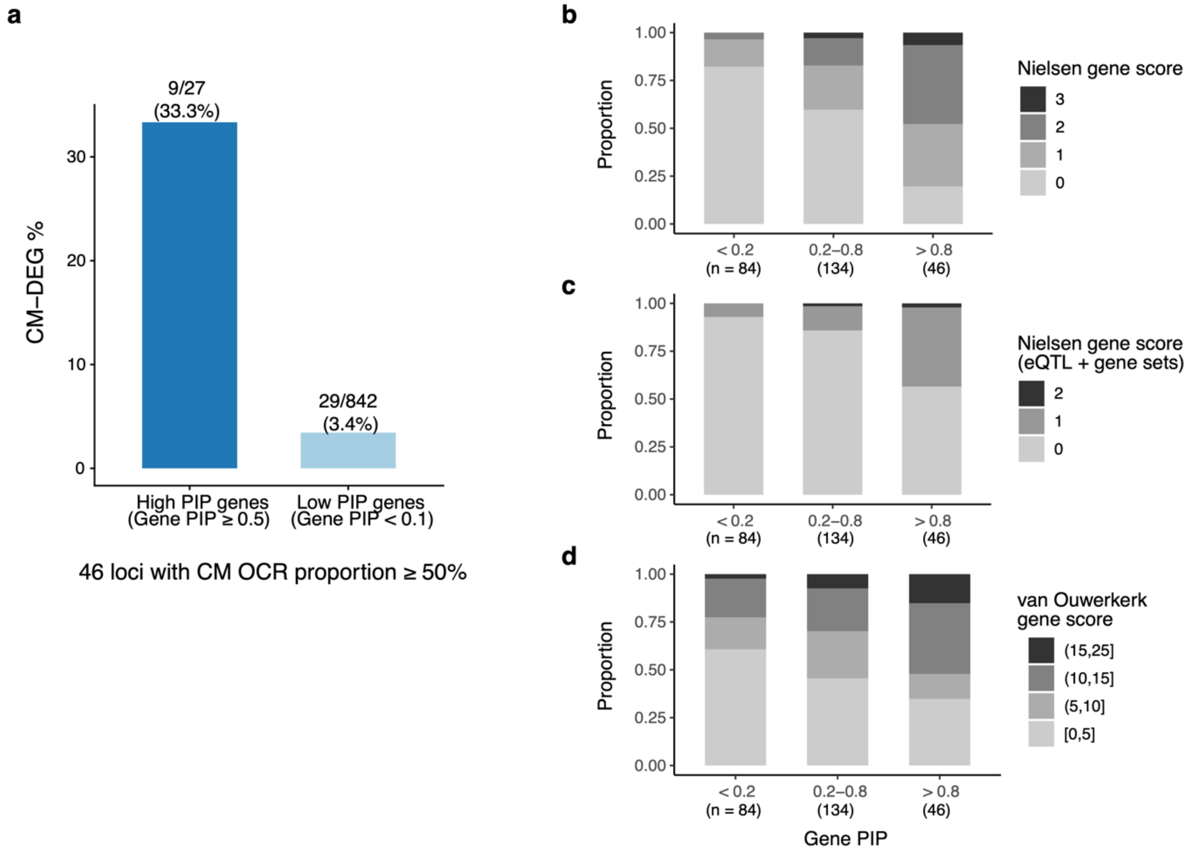

Supplementary Fig. 13: a, Enrichment of CM DEGs in high PIP genes vs. low PIP genes in loci with high CM OCR proportion. **b,c,d, Support of high confidence genes (gene PIP >= 0.8) from functional data collected in earlier AF studies. b,** The proportions of genes at different gene PIP bins with a functional score (0-3) assigned by Nielsen *et al.*[2] The score of a gene was calculated from four sources of information about the GWAS lead SNP and the gene: distance of SNP to gene, exonic variant, eQTL and gene sets. A gene thus has a score of 0-4, with each category contributing a score of 1. **c**, as in (**b**) but the scores were calculated based only on eQTL and gene set annotations. This is to reduce the bias that was introduced in computing gene PIPs, i.e. genes with exonic variants and closest to GWAS SNPs tend to have higher PIPs. **d**, as (**b**) but using gene scores developed in van Ouwerkerk *et al.*[3] Genes that are in the same Topologically Associating Domains (TADs) of the GWAS SNPs, eQTL targets, and expressed in heart tissues across multiple datasets have high scores.

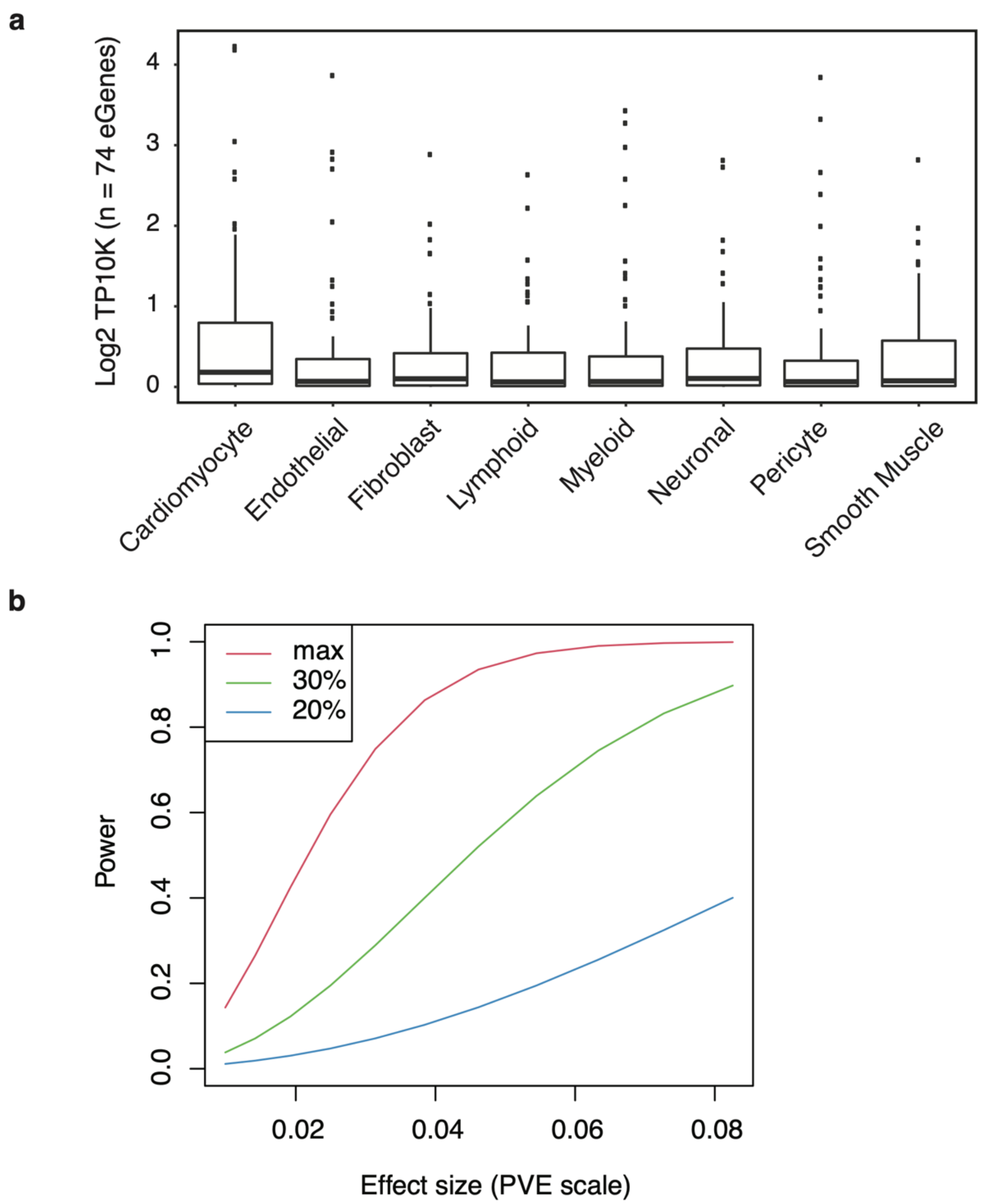

Supplementary Fig. 14: Tissue sharing analysis of eQTLs. a, Expression levels of 74 eGenes associated with CM-specific OCRs in heart cell types, from scRNA-seq. See Supplementary Notes. The center line of a box represents the median; the lower and upper hinges of a box correspond to the first and third quartiles; the upper/lower whisker extends from the hinge to the largest/smallest value no further than 1.5 * inter-quartile range from the hinge. **b**, Power of detecting cell-type-specific eQTLs in bulk samples, as function of effect size. Simulated are 3 scenarios reflecting different proportions with which a cell type occurs in the bulk tissue (1, 0.5, 0.3). The effect size of an eQTL is defined at the PVE scale, i.e. the proportion of variance of gene expression explained by the genetic variation at that eQTL.

Supplementary Notes

1 Power analysis of bulk eQTL detection

Most eQTL studies were performed using bulk samples, which are mix of multiple cell types. Intuitively, eQTLs with effects in common cell types are easier to detect, so are those with effects shared across multiple cell types. We will quantitatively analyze how the power of bulk eQTL study depends on cell type compositions and effect sizes across cell types. A primary goal is to understand how the power of detecting eQTLs acting on single cell types is reduced, compared to the eQTLs with shared effects across cell types.

Let's consider one SNP-gene pair a time. Let y_i be the total expression, across K cell types, of the gene in sample i, and G_i be the genotype of sample i, $1 \leq i \leq n$. Also let p_{ik} be the proportion of cell type $k, 1 \leq k \leq K$ in sample i, with $\sum_k p_{ik} = 1$. The total expression y_i is the sum of expression across K cell types, weighted by the cell type proportions. Let y_{ik} be expression of the gene in cell type k in the sample i, we then have:

$$y_i = \sum_k p_{ik} y_{ik}. \tag{1}$$

To relate gene expression with the genotype, we denote β_k, the effect size of the SNP on gene expression in cell type k, which may vary across cell types. Thus for a cell type k, we have

$$y_{ik} = \mu_k + G_i \beta_k + \epsilon_{ik} \qquad \epsilon_{ik} \sim N(0, \sigma_k^2), \tag{2}$$

where μ_k is the average expression in cell type k, and σ_k its standard deviation. Summing over all cell types, we have the following model of the bulk gene expression in sample i:

$$y_i = \sum_k p_{ik}\mu_k + G_i \sum_k p_{ik}\beta_k + \sum_k p_{ik}\epsilon_{ik} \qquad \epsilon_{ik} \sim N(0, \sigma_k^2). \tag{3}$$

To gain some intuitions of how the power may depend on various parameters, we make the simplifying assumption that cell type compositions are similar across samples, i.e. $p_{ik} \approx p_k$, for all i. We now have:

$$y_i = \mu + G_i \sum_k p_k \beta_k + \sum_k p_k \epsilon_{ik} \qquad \epsilon_{ik} \sim N(0, \sigma_k^2), \tag{4}$$

where $\mu = \sum_k p_k \mu_k$ is the average gene expression. Comparing this with regression equation in standard eQTL mapping, we see that the effective bulk eQTL effect is simply $\tilde{\beta} = \sum_k p_k \beta_k$. Now we can see that if an eQTL is specific to a single cell type, say, k, then the "effective" eQTL effect size becomes $p_k \beta_k$. Clearly, the reduction of effect size is large for rare cell types.

While the change of effect size is straightforward, the problem is complicated by the fact that the power of mapping eQTL depends not only on the effect size, but also the total variance of gene expression. In fact, the power is determined by the sample size and the percent of variance of gene expression explained (PVE) by the genetic variant. The total variance, and hence PVE, according to the model above, depend on the variance of the error term, σ_k's, and also on how the errors across cell types may be correlated. Below, we consider several simplified cases, with the goal of comparing power of detecting cell-type shared vs. cell-type specific eQTLs.

We first consider a special case where the eQTL effects are shared across all cell types, i.e. $\beta_k = \beta$ for all cell type k. Furthermore, the gene expression have the same variance, and are fully correlated across all

cell types. Under this "full sharing" scenario, an eQTL would have exactly the same effect across all cell types. Thus the PVE of the variant on any cell type would be the same as the PVE in the bulk sample. As a result, the power of detecting this shared eQTL would be the same as the power when we have expression data from pure cell types.

Next we consider the case where eQTL is cell type-specific. Suppose $\beta_c \neq 0$ for some cell type c, and 0 for all other ones. The effect size in the bulk data is then $\tilde{\beta} = p_c \beta_c$. As explained above, the actual power would depend on expression in other cell types. In the special case where the gene is expressed only in cell type c, then the variance of gene expression in other cell types would be 0. The residual variance of gene expression (after considering eQTL effect) is then:

$$\mathrm{Var}\left(\sum_k p_k \epsilon_{ik}\right) = p_c^2 \sigma_c^2. \tag{5}$$

Assuming genotype G_i is standardized i.e. its variance is equal to 1, the PVE of the eQTL on the bulk gene expression is then:

$$\mathrm{PVE} = \frac{p_c^2 \beta_c^2}{p_c^2 \beta_c^2 + p_c^2 \sigma_c^2} = \frac{\beta_c^2}{\beta_c^2 + \sigma_c^2}, \tag{6}$$

which is exactly the PVE of the eQTL in the cell type c alone. This analysis thus suggests that there is no power loss of detecting cell-type specific eQTLs when gene expression is limited to that cell type.

In practice, however, even if an eQTL is specific to a cell type, the associated gene may be expressed much more broadly. Using our scRNA-seq data, we assessed the expression pattern of the eGenes detected in GTEx heart whose eQTLs are fine-mapped to Cardiomyocyte (CM)-specific OCRs. While the eQTL effects are likely CM-specific, the expression of genes are generally not restricted to CMs. In fact, the distributions of expression levels of these genes overlap considerably across cell types (Supplementary Fig. 14a). For example, the median gene expression in CMs corresponds to top 34% expression of endothelial genes, and 38% of fibroblast. Motivated by this observation, and for mathematical simplicity, we assume equal expression variance across cell types, i.e. $\sigma_k^2 = \sigma^2$. Furthermore, we assume the errors are uncorrelated across cell types. Under these assumptions, the PVE of the eQTL on bulk gene expression is given by:

$$\mathrm{PVE} = \frac{p_c^2 \beta_c^2}{p_c^2 \beta_c^2 + \sigma^2 \sum_k p_k^2}. \tag{7}$$

With out loss of generality, we assume $\sigma^2 = 1$, in other words, our effect sizes are measured at the scale/unit of σ. Now given p_k of all cell types, and β_c, we can evaluate PVE. To assess the power, we use this simple relationship from standard linear regression: let $\hat{z}$ be the Z-score of the association test, n sample size, then $\hat{z} \sim N(\sqrt{n \cdot \mathrm{PVE}}, 1)$.

In our simulations, we use cell type proportions similar to those in real data. We have 7 cell types with proportions 0.3, 0.2, 0.2, 0.1, 0.1, 0.05 and 0.05, respectively. We consider two cases $p_c = 0.3$ and 0.2, respectively. We then vary β_c to assess PVE and then power at $p < 10^{-3}$, using sample size of $n = 500$. To assess how much power is lost from bulk samples, compared with the case where we have "pure" cell type of c, we evaluate the power under

$$\mathrm{PVE}_{\max} = \frac{\beta_c^2}{\beta_c^2 + \sigma^2} = \frac{\beta_c^2}{\beta_c^2 + 1}. \tag{8}$$

Our simulation shows that the power of detecting cell-type specific eQTLs, especially when cell type proportion is low, is substantially reduced (Supplementary Fig. 14b).

We note that our assumption of independent gene expression across cell types is probably overly-simplified. In reality, gene expression are likely positively correlated across cell types. Ignoring this correlation most likely leads to under-estimation of the total residual variance of gene expression. For a given eQTL effect size, this under-estimation would lead to over-estimated PVE, and hence power. So the power loss for cell-type specific eQTLs is likely even larger than our simulations showed.

In conclusion, our analysis suggests that the power of detecting cell-type specific eQTLs is likely low in bulk-eQTL studies, and this probably contributes to the observed high level of eQTL sharing across tissues, as well as the observation that the bulk eQTLs explain low heritability of complex traits.

Supplementary References

1. Litviňuková, M. et al. Cells of the adult human heart. *Nature* **588**, 466–472 (2020).

2. Nielsen, J. B. et al. Biobank-driven genomic discovery yields new insight into atrial fibrillation biology. *Nat. Genet.* **50**, 1234–1239 (2018).

3. Ouwerkerk, A. F. van et al. Identification of atrial fibrillation associated genes and functional non-coding variants. *Nat. Commun*. **10**, 4755 (2019).
