# Supplementary material for: Single-cell genomics improves the discovery of risk variants and genes of atrial fibrillation
Source: Nat Commun. 2023 Aug 17;14:4999. doi: 10.1038/s41467-023-40505-5 (PMC10435551; doi:10.1038/s41467-023-40505-5)
Supplement: Supplementary file 3 — Description of Additional Supplementary Files [file 41467_2023_40505_MOESM3_ESM.pdf]

Description of Additional Supplementary Files

File Name: Supplementary Data 1
Description: Meta-data of the heart samples used in the study.

File Name: Supplementary Data 2
Description: TF enrichment in different cell types.

File Name: Supplementary Data 3
Description: Results of SNP fine-mapping analysis, and gene mapping.

File Name: Supplementary Data 4
Description: Comparing high PIP variants with variants with allele-specific activitiy using STARR-seq from van Ouwerkerk *et al.* 2020.

File Name: Supplementary Data 5
Description: Sum of PIPs for SNPs assigned to functional categories.

File Name: Supplementary Data 6
Description: SNPs chosen for experimental study.

File Name: Supplementary Data 7
Description: Colocalization result of fine-mapped SNPs with eQTLs.

File Name: Supplementary Data 8
Description: Gene mapping results, summarized at the gene level.

File Name: Supplementary Data 9
Description: Gene mapping results, summarized at the level of LD blocks.

File Name: Supplementary Data 10
Description: Information of genes at PIP >= 0.8 with literature evidence.

File Name: Supplementary Data 11
Description: Loci where the mapped high PIP (>= 0.8) genes are not the nearest genes.

File Name: Supplementary Data 12
Description: OMIM gene information.

File Name: Supplementary Data 13
Description: GO enrichment of genes with gene PIP >= 0.8.

File Name: Supplementary Data 14
Description: GO enrichment of genes with gene PIP >= 0.5 (from STRING).

File Name: Supplementary Data 15
Description: High PIP eQTLs and their categories.
