# Supplementary material for: Single-cell genomics improves the discovery of risk variants and genes of atrial fibrillation
Source: Nat Commun. 2023 Aug 17;14:4999. doi: 10.1038/s41467-023-40505-5 (PMC10435551; doi:10.1038/s41467-023-40505-5)
Supplement: Supplementary file 5 — Reporting Summary [file 41467_2023_40505_MOESM5_ESM.pdf]

nature portfolio

Corresponding author(s): Sebastian Pott, Xin He, Anindita Basu

Last updated by author(s): Aug 9, 2023

Reporting Summary

Nature Portfolio wishes to improve the reproducibility of the work that we publish. This form provides structure for consistency and transparency in reporting. For further information on Nature Portfolio policies, see our Editorial Policies and the Editorial Policy Checklist.

Statistics

For all statistical analyses, confirm that the following items are present in the figure legend, table legend, main text, or Methods section.

n/a	Confirmed	
☐	☒	The exact sample size (*n*) for each experimental group/condition, given as a discrete number and unit of measurement
☐	☒	A statement on whether measurements were taken from distinct samples or whether the same sample was measured repeatedly
☐	☒	The statistical test(s) used AND whether they are one- or two-sided<br>*Only common tests should be described solely by name; describe more complex techniques in the Methods section.*
☐	☒	A description of all covariates tested
☐	☒	A description of any assumptions or corrections, such as tests of normality and adjustment for multiple comparisons
☐	☒	A full description of the statistical parameters including central tendency (e.g. means) or other basic estimates (e.g. regression coefficient) AND variation (e.g. standard deviation) or associated estimates of uncertainty (e.g. confidence intervals)
☐	☒	For null hypothesis testing, the test statistic (e.g. *F*, *t*, *r*) with confidence intervals, effect sizes, degrees of freedom and *P* value noted<br>*Give P values as exact values whenever suitable.*
☐	☒	For Bayesian analysis, information on the choice of priors and Markov chain Monte Carlo settings
☒	☐	For hierarchical and complex designs, identification of the appropriate level for tests and full reporting of outcomes
☐	☒	Estimates of effect sizes (e.g. Cohen's *d*, Pearson's *r*), indicating how they were calculated

Our web collection on statistics for biologists contains articles on many of the points above.

Software and code

Policy information about availability of computer code

Data collection	No software was used for data collection.
Data analysis	Code for data processing and analyses are available at https://github.com/xinhe-lab/aFib_heart_atlas_mapgen_paper. The Mapgen R package is available from https://github.com/xinhe-lab/ mapgen. In addition, raw sequencing data for snRNA-seq were processed and aligned with STARsolo 2.6.1 using a custom wrapper script that can be accessed here: https://github.com/aselewa/dropseqRunner. scATAC-seq data were processed with CellRanger-atac 1.2.0

For manuscripts utilizing custom algorithms or software that are central to the research but not yet described in published literature, software must be made available to editors and reviewers. We strongly encourage code deposition in a community repository (e.g. GitHub). See the Nature Portfolio guidelines for submitting code & software for further information.

Data

Policy information about availability of data

All manuscripts must include a data availability statement. This statement should provide the following information, where applicable:

- Accession codes, unique identifiers, or web links for publicly available datasets
- A description of any restrictions on data availability
- For clinical datasets or third party data, please ensure that the statement adheres to our policy

snRNA-seq and scATAC-seq data has been deposited in GEO repository: accession GSE224997.

Research involving human participants, their data, or biological material

Policy information about studies with human participants or human data. See also policy information about sex, gender (identity/presentation), and sexual orientation and race, ethnicity and racism.

Reporting on sex and gender	We included heart tissue samples from 3 male individuals.
Reporting on race, ethnicity, or other socially relevant groupings	n/a
Population characteristics	n/a
Recruitment	n/a
Ethics oversight	University of Chicago IRB reviewed the study and determined that this research does not constitute Human Subjects Research (IRB19-1429, 9/6/2019)

Note that full information on the approval of the study protocol must also be provided in the manuscript.

Field-specific reporting

Please select the one below that is the best fit for your research. If you are not sure, read the appropriate sections before making your selection.

☒ Life sciences ☐ Behavioural & social sciences ☐ Ecological, evolutionary & environmental sciences

For a reference copy of the document with all sections, see nature.com/documents/nr-reporting-summary-flat.pdf

Life sciences study design

All studies must disclose on these points even when the disclosure is negative.

Sample size	Heart tissue samples were obtained from National Disease Research Interchange (NDRI). We included samples from 4 regions (left and right ventricles, interventricular septum, apex) from 3 male individuals.
Data exclusions	data analysis was performed following standard procedures for single cell RNA-seq and single cell ATAC-seq and all filter criteria are documented in the Method section and shared code
Replication	3 biological replicates (individuals) from 4 regions which were analysed together
Randomization	no randomization was performed
Blinding	Investigators were not blinded to individual or location labels

Reporting for specific materials, systems and methods

We require information from authors about some types of materials, experimental systems and methods used in many studies. Here, indicate whether each material, system or method listed is relevant to your study. If you are not sure if a list item applies to your research, read the appropriate section before selecting a response.

Materials & experimental systems

n/a	Involved in the study	
☒	☐	Antibodies
☐	☒	Eukaryotic cell lines
☒	☐	Palaeontology and archaeology
☒	☐	Animals and other organisms
☒	☐	Clinical data
☒	☐	Dual use research of concern
☒	☐	Plants

Methods

n/a	Involved in the study	
☒	☐	ChIP-seq
☒	☐	Flow cytometry
☒	☐	MRI-based neuroimaging

Eukaryotic cell lines

Policy information about cell lines and Sex and Gender in Research

Cell line source(s)	HL-1 were received from Claycomb WC. Reference: Neilan CL, Kenyon E, Kovach MA, Bowden K, Claycomb WC, Traynor JR, Bolling SF. An immortalized myocyte cell line, HL-1, expresses a functional delta -opioid receptor. Journal of Molecular and Cellular Cardiology. 2000;32:2187–2193. doi: 10.1006/jmcc.2000.1241. HL-1 cells were originally derived from an AT-1 atrial cardiomyocyte tumor from an adult female C57BL/6J mouse as described in Claycomb et al, 1998.<br>3T3 Embryonic mouse fibroblasts were obtained from ThermoFisher.
Authentication	Original authentication and validation in Claycomb et al, 1998, the authors authenticate this line through gene expression analyses, immunohistochemistry, and electrophysiology approaches which found the HL-1 cell line behaves similarly to adult atrial myocytes. No authentication of either cell line was performed after obtained by our group
Mycoplasma contamination	Not tested
Commonly misidentified lines<br>(See ICLAC register)	HL-1 and 3T3 are lines not listed in the commonly misidentified cell line database
